# Myocardial contractility is preserved early but reduced late after ovariectomy in young female rats

**DOI:** 10.1186/1477-7827-9-54

**Published:** 2011-04-23

**Authors:** Altemar S Paigel, Eduardo Hertel Ribeiro, Aurelia A Fernandes, Gabriel P Targueta, Dalton V Vassallo, Ivanita Stefanon

**Affiliations:** Department of Physiological Sciences, Federal University of Espirito Santo, Vitória, ES Brazil

**Keywords:** Sarcoplasmic Reticulum, Myocardial Contractility, Cardiac Contractility, Inotropic Response, Cardiac Sarcoplasmic Reticulum

## Abstract

**Background:**

Ovarian sex hormones (OSHs) are implicated in cardiovascular function. It has been shown that OSHs play an important role in the long term regulation of cardiac sarcoplasmic reticulum (SR) function and contractility, although early effects of OSHs deprivation on myocardial contractility have not yet been determined. This study evaluated the early and late effects of OSHs deficiency on left ventricular contractility in rats after ovariectomy.

**Methods:**

Young female Wistar rats were divided into 3 groups (n = 9-15): sham operated (Sham), ovariectomized (Ovx) and Ovx treated with estradiol (1 mg/kg, i.m., once a week) (Ovx+E2). After 7, 15, 30 and 60 days post Ovx, left ventricle papillary muscle was mounted for isometric tension recordings. The inotropic response to Ca2+ (0.62 to 3.75 mM) and isoproterenol (Iso 10-8 to 10-2 M) and contractility changes in response to rate changes (0.25 to 3 Hz) were assessed. Protein expression of SR Ca2+-ATPase (SERCA2a) and phospholamban (PLB) in the heart was also examined.

**Results:**

The positive inotropic response to Ca2+ and Iso at 7, 15, and 30 days after Ovx was preserved. However, at 60 days, the Ovx group had decreased myocardial contractility which was subsequently restored with E2 replacement. The reduction in SERCA2a and increase in PLB expression observed at 60 days after Ovx were restored with E2 replacement.

**Conclusion:**

This study demonstrated that myocardial contractility and expression of key Ca2+ handling proteins were preserved in the early phase and reduced at long-term during OSHs deprivation.

**Electronic supplementary material:**

The online version of this article (doi:10.1186/1477-7827-9-54) contains supplementary material, which is available to authorized users.

## Background

Cardiovascular disease is a major contributor to morbidity and mortality among post-menopausal women in the western world [1]. Interestingly, pre-menopausal women have a reduced risk of mortality from cardiovascular disease while post-menopausal women have a similar or even increased risk for cardiovascular disease as compared to men [2]. It has been suggested that ovarian sex hormones (OSHs) have a protective effect on the cardiovascular system [2, 3]. However, the effects of estrogen replacement therapy on the cardiovascular risk only account for about 50% of the reduction seen in cardiovascular disease suggesting that there must be additional mechanisms whereby estrogen exerts its cardioprotective effects [4]. Though observational and experimental studies have suggested that ovarian sex hormones (OSHs) replacement therapy may be cardioprotective [5, 6], large trials utilizing oral conjugated estrogen and medroxyprogesterone acetate did not confirm these findings [7].

In the last few years, the main ovarian hormone, estrogen (E2), has received special attention for its protective effects against atherosclerosis [8, 9]. Several studies have also indicated that E2 directly protective of cardiac muscle contractility [10, 11]. However, though studies have indicated that OSHs therapy restore contractile performance in post-menopausal women [12–14], short-term of cardiac effects of ovariectomy and E2 replacement have not been demonstrated.

Recent studies further suggest that mechanical functioning and proteomic profiles in ventricular myocytes are directly regulated by E2 [15–18]. However, those studies only investigated cardiac performance and proteins involved in Ca^2+^ homeostasis at 4 to 10 weeks after OSHs deprivation leaving short-term effects of OSHs deprivation on myocardial contractility poorly understood. Thus, our aim was to study the inotropic response of left ventricular papillary muscles short and long-term (7, 15, 30 and 60 days) after ovariectomy in young female rats. We also investigated the expression of cardiac regulatory proteins sarcoplasmic reticulum (SR) Ca^2+^-ATPase (SERCA2a), phospholamban (PLB) and sodium-calcium exchanger (NCX) at 7, 15, 30 and 60 days after Ovx. We studied the expression of these proteins primarily due the fact that PLB regulates the Ca^2+^-affinity of SERCA2a, facilitating calcium uptake into the SR and muscle relaxation, which is considered an important intracellular mechanism involved in myocardial contractility [19, 20].

## Methods

### Animal care

The care and use of the laboratory animals were in accordance with National Institutes of Health (NIH) guidelines and were approved by the local animal ethics committee. All rats had free access to water and were fed with food ad libitum.

Experimental groups, surgical procedures and E2 replacement. Eight-week-old female Wistar rats weighting 185g approximately were randomly divided into two groups. One group underwent bilateral ovariectomy (Ovx) as described previously [21]. Briefly, a dorsal midline skin incision was made under anesthesia caudal to the posterior border of the ribs. The posterior abdominal muscle wall was bluntly dissected, the abdominal cavity was opened and the ovary was gently exteriorized and removed. The uterine horn was returned into the abdomen. The skin incision was closed with sterile nylon sutures, and the process was repeated on the other side. At 1 week after Ovx, a subgroup of the ovariectomized rats (Ovx+E2) started treatment with estradiol valerate (E2) 1 mg/Kg injected, i.m., once a week for 8 weeks. The second group underwent a sham operation and served as normal controls (sham). Left ventricle papillary muscle contractility was studied at 7, 15, 30 and 60 days after surgery. E2 replacement was carried out only in the 60 day ovariectomized subgroup because it was the only group that showed a reduction in myocardial contractility. At the time of sacrifice, adequacy of the ovariectomy was determined grossly by the absence of ovarian tissue and marked atrophy of the uterus [16]. We also determined the weight of the entire animal, as well as the weight of the left and right ventricles and the uterus.

### Isometric tension and myocardial contractility

Rats received 500 units of heparin intraperitoneal (i.p.) and then were anesthetized 10 minutes later with 45 mg/kg sodium pentobarbital, i.p., (Cristália, SP, Brazil). Hearts were rapidly removed and perfused through the aortic stump and the left ventricle papillary muscles were dissected. Muscle preparations were mounted for isometric tension recording and maintained in a 20 mL glass-bath containing Krebs-Henseleit solution (in mM: NaCl 118, KCl 4.7, CaCl_2_ 1.25, KH_2_PO_4_ 1.2, MgSO_4_ 1.2, NaHCO_3_ 23 and glucose 11) at 30°C and pH = 7.4, which was continuously aerated with 95% O_2_ and 5% CO_2_. Resting tension was adjusted to produce maximal contractile force (Lmax). The twitch contraction rate was controlled by isolated rectangular pulses (10 to 15 V, 12 ms duration) through a pair of platinum electrodes. The standard stimulation rate was 0.5 Hz (steady-state). Isometric force development was measured with an isometric force transducer (TSD105A, Biopac) and normalized to muscle weight (g/g). Recording started after 60 minutes to permit the muscle to adapt to the new environmental conditions. Myocardial contractility was tested by the following protocol. First, we measured the inotropic response to changes in extracellular calcium concentration (Ca^2+^, 0.62 to 3.75 mM). Next, the positive inotropic response, produced by increasing isoproterenol concentrations added to the bath (10^-8^ - 10^-2^ M), was analyzed. Finally, we evaluated the isometric force development relationship at stimulation rates of 0.25 to 3.0 Hz. At the end of the experiment, the papillary muscle was removed and weighed for force normalization (g/g).

### Serum estrogen level

Blood samples were collected in tubes with anticoagulant from group at 60 days after surgery. Blood samples were centrifuged at 1,500 *g* for 15 min at 4°C. The resulting serum was kept at -80°C until used to determine estrogen levels. The E_2_ levels in the serum were measured using ELISA technique according to the manufacturer's instruction.

### Western blot analysis of SERCA2a, NCX and PLB

Proteins from homogenized hearts (100 μg) were separated by 7.5% (SERCA2a and NCX) or 15% (PLB) SDS-PAGE. Proteins were transferred to nitrocellulose membranes for SERCA2a and NCX and polyvinyl difluoride membranes for PLB and were incubated with mouse monoclonal antibodies for SERCA2a (1:1000, Affinity BioReagents, CO, USA), NCX (1:200, Abcam Cambridge, MA, USA) and PLB (1 μg/ml, Affinity BioReagents, CO, USA). After washing, membranes were incubated with anti-mouse (1:5000, StressGen, Victoria, Canada) immunoglobulin antibody conjugated to horseradish peroxidase. After thorough washing, immunocomplexes were detected using an enhanced horseradish peroxidase/luminal chemiluminescence system (ECL Plus, Amersham International, Little Chalfont, UK) and film (Hyperfilm ECL International). Signals on the immunoblot were quantified with the National Institutes of Health Image V1.56 computer program. The same membrane was used to determine GAPDH expression using a mouse monoclonal antibody (1:5000, Abcam Cambridge, MA, USA).

### Statistical analysis

All values are expressed as mean ± SEM. Differences among groups were analyzed using the two way ANOVA test followed by the Tukey post hoc test for multiple comparisons. A p value < 0.05 was considered statistically significant. For protein expression, data are expressed as the ratio between protein and GAPDH signals.

### Drugs and chemicals

All chemicals, unless specified in the text, were purchased from Sigma Chemical (St. Louis, MO).

## Results

### Rat weights

The body weights of the ovariectomized rats were significantly greater than those of sham controls at 15, 30 and 60 days (Table 1). E2 treatment prevented weight gain at 60 days after ovariectomy and also restored the estrogen levels at the same levels as the control animals (Sham: 127 ± 11.5 pmol/L, Ovx : 50.4 ± 11.4* pmol/L and Ovx + E2: 142.2 ± 31 pmol/L; p < 0.05). Ovariectomy and E2 replacement did not alter the left or right ventricle weight to body weight ratio. Starting at 7 days, the deficiency of OSH induced a significant decrease in uterine weight compared with that of sham controls. Restoration of uterine mass was observed in the E2 treated group (Table 1).

**Table 1:** Weight data from sham, ovariectomized (Ovx) and estrogen replacement

	7 days		15 days		30 days		60 days		
	Sham	Ovx	Sham	Ovx	Sham	Ovx	Sham	Ovx	Ovx+E2
**BW (g)**	195 ± 3	201 ± 5	204 ± 3	232 ± 8*	233 ± 3	274 ± 5*	256 ± 5	310 ± 9*	251 ± 9^#^
**LV/BW (mg/g)**	2.25 ± 0.06	2.12 ± 0.07	2.21 ± 0.07	2.07 ± 0.05	2.25 ± 0.09	2.09 ± 0.06	2.0 ± 0.03	1.9 ± 0.04	1.93 ± 0.05
**RV/BW (mg/g)**	0.57 ± 0.02	0.55 ± 0.02	0.52 ± 0.02	0.53 ± 0.01	0.54 ± 0.01	0.58 ± 0.04	0.48 ± 0.03	0.49 ± 0.02	0.54 ± 0.02
**Uterus/BW (mg/g)**	2.09 ± 0.14	0.70 ± 0.04*	2.40 ± 0.17	0.60 ± 0.05*	1.96 ± 0.07	0.47 ± 0.04*	2.60 ± 0.16	0.45 ± 0.04*	2.56 ± 0.19^#^
**Papillary Muscle (mg)**	5.5 ± 0.46	5.6 ± 0.56	4.9 ± 0.35	6.1 ± 0.51	4.11 ± 0.36	5.0 ± 0.4	5.2 ± 0.47	5.7 ± 0.33	5.9 ± 0.5

LV - Left Ventricle, RV - Right Ventricle, BW - Body Weight. Data are expressed as mean ± SEM.

*OVx vs Sham, ^#^ Ovx+E2 vs Ovx, p < 0.05 unpaired t-test. N = 6-15 (Ovx+E2) groups

### Isometric contractility response to calcium

**Figure 1:**
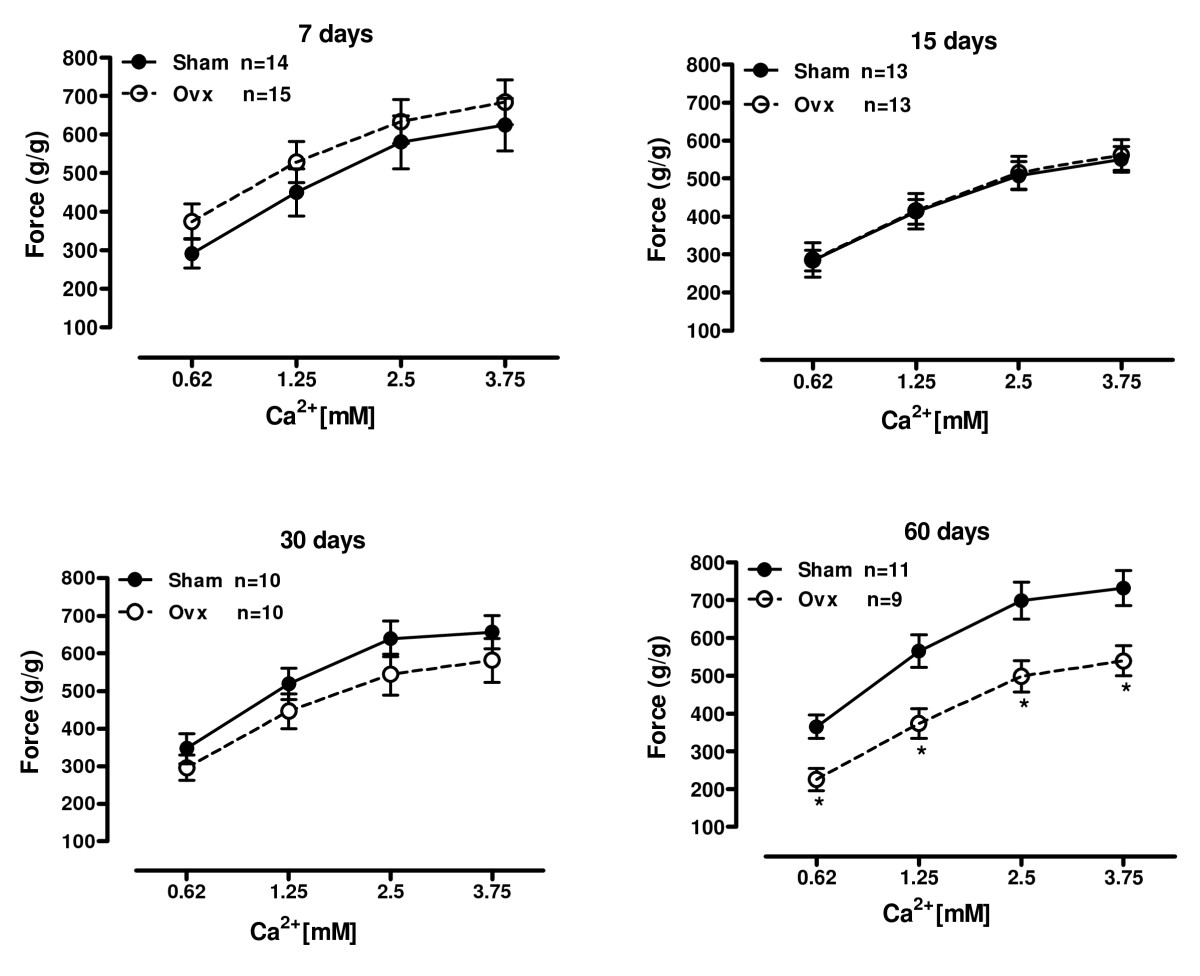
**Inotropic response to calcium**. Effect of increasing extracellular calcium concentration from 0.62 to 3.75 mM on force development in isolated left ventricle papillary muscles from sham operation (Sham) and ovariectomized (Ovx) rats at 7, 15, 30 and 60 days after surgery. Force (g/g) values are expressed as mean ± S.E.M. *different from sham (*P* < 0.05) using two-way ANOVA and post hoc Tukey test. Number of animals used is indicated.

It is well known increases in extracellular Ca^2+^ results in enhanced calcium influx through L-type Ca^2+^ channels triggering further Ca^2+^ release from Ca^2+^ stores within the SR [22]. This in turn leads to a rise of intracellular free Ca^2+^ culminating in myofilament shortening and cell contraction. As expected, increases in extracellular Ca^2+^ resulted in a positive inotropic response (Figure 1). We found no significant differences in the inotropic response to calcium at 7, 15, and 30 days among ovariectomy and control groups. However, deprivation of OSHs at 60 days induced a reduction in the inotropic response to extracellular Ca^2+^.

Force increases with increasing myocardial mass, characterizing a positive correlation between theses variables. Assuming the 60 days group is older and the papillary muscles are heavier, we expected to find higher values for all parameter in the 60 days groups. As the OVX group has a contractility dysfunction, the force did not increase as expected.

### Isometric contractile response to β-adrenergic receptor stimulation

**Figure 2:**
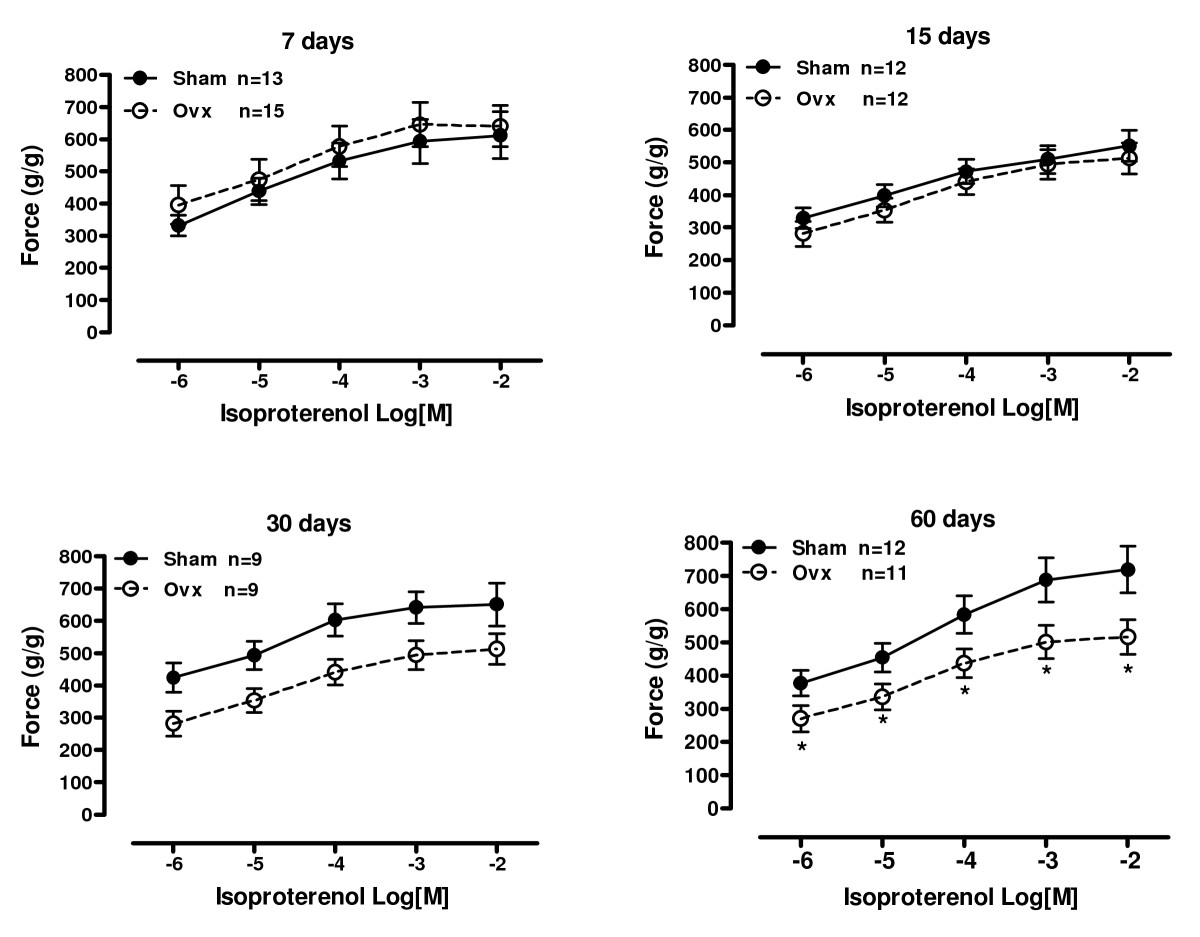
**Inotropic response to isoproterenol**. Effect of the β-adrenergic agonist isoproterenol in increasing concentrations from 10^-8^ to 10^-2^ M on force development in isolated left ventricle papillary muscles from Sham operation (Sham) and ovariectomized (Ovx) rats at 7, 15, 30 and 60 days after surgery. Extracellular Ca^2+^ concentration was 0.62 mM. Force (g/g) values are expressed as mean ± S.E.M. *different from sham (*P* < 0.05) using two-way ANOVA and post hoc Tukey test. Number of animals used is indicated.

Using a nonspecific β-adrenergic agonist, isoproterenol, dose-response curves were assessed (Figure 2). Stimulation of this subtype of β-adrenergic receptor in the heart by isoproterenol increases contractility and accelerates relaxation by activating the adenylyl cyclase/cAMP/protein kinase pathway [23]. As expected, isoproterenol promoted a positive inotropic effect in all groups examined. However, the positive inotropic response was decreased in the ovariectomized group at 60 days compared with sham controls.

### Force-frequency relationship

**Figure 3:**
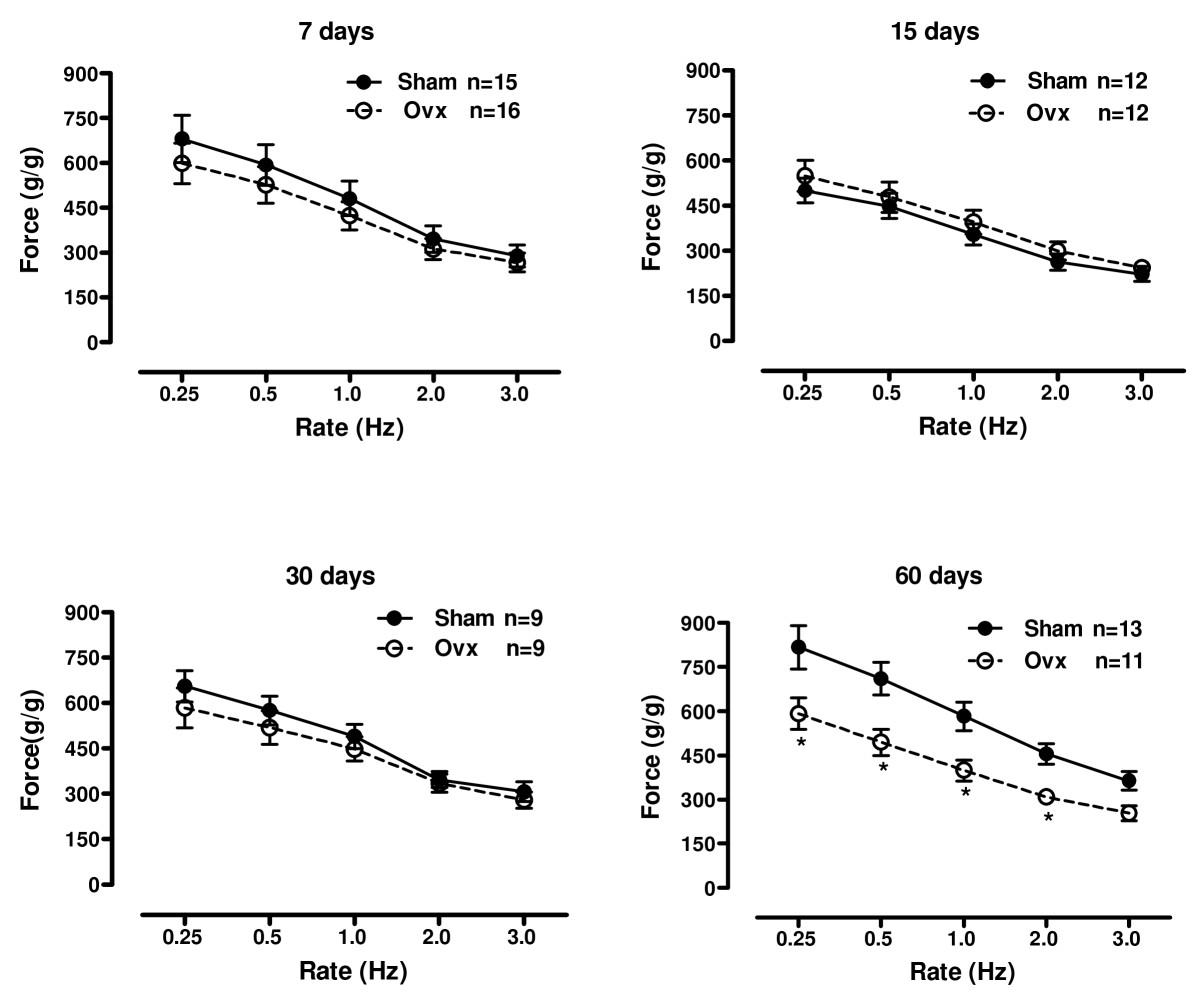
**Inotropic response to frequence**. Effect of ovariectomy on the rate-isometric force development relationship from 0.25 to 3.0 Hz in isolated left ventricle papillary muscles from sham operation (Sham) and ovariectomized (Ovx) rats at 7, 15, 30 and 60 days after surgery. Force (g/g) values are expressed as mean ± S.E.M. *different from sham (*P* < 0.05) using two-way ANOVA and post hoc Tukey test. Number of animals used is indicated.

The relationship between rate and isometric force (Figure 3) is species-specific and was first described by Bowditch in 1871. In the rat, a reverse staircase phenomenon has been demonstrated, where force of contraction falls as heart rate increases. It has been demonstrated that the time to peak isometric force varies directly with the duration of the active state, and thus changes in duration are reflected in changes in the peak force [24]. The Bowditch staircase phenomenon was investigated by changing the stimulation rate from 0.25 to 3.0 Hz following rest periods of approximately 60 s. As expected, a decrease in rate of stimulation produced an immediate increase in peak force. This inverse relationship was preserved in all groups, and was similar between controls and Ovx rats at 7, 15 and 30 days but reduced in Ovx rats at 60 days.

### Effect of ovariectomy on maximum positive and negative force derivatives

**Figure 4:**
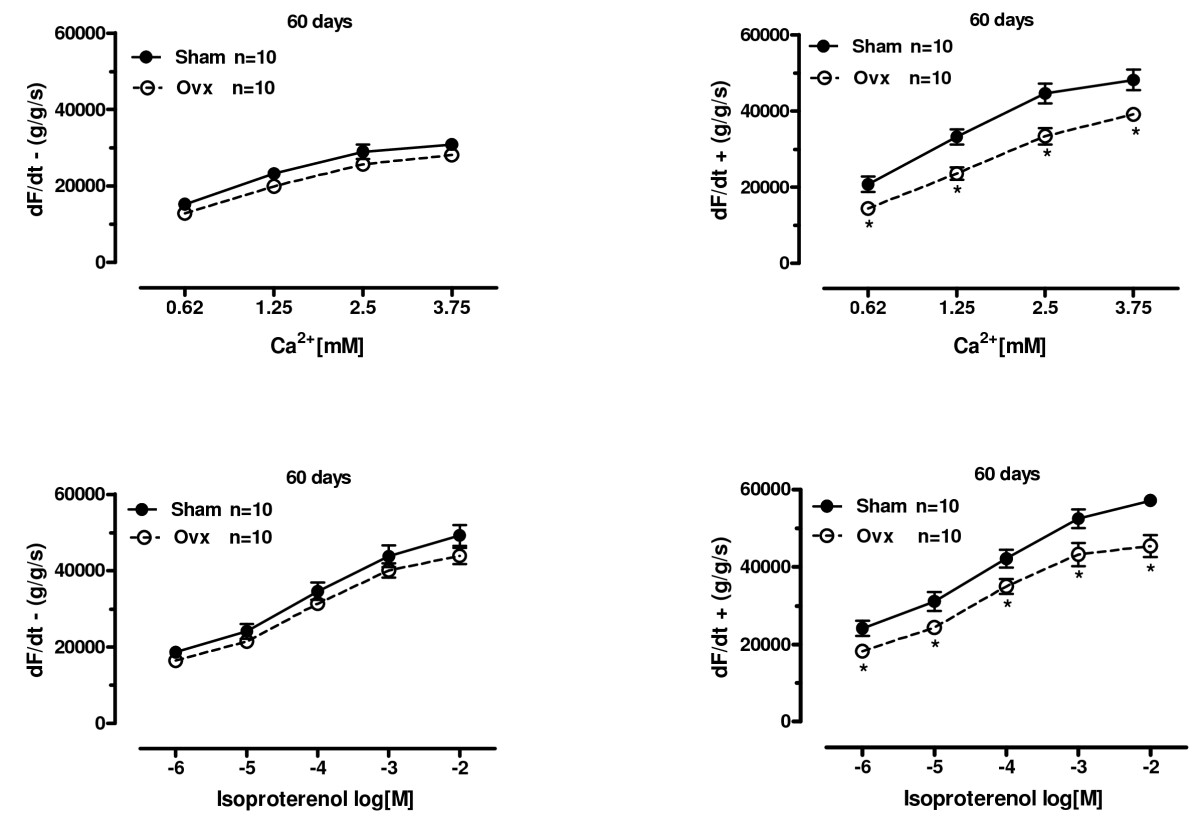
**Lusitropic response to calcium and isoproterenol**. Effect of ovariectomy on maximal and minimal rate of change in force in left ventricle papillary muscles isolated from sham operation (Sham) and ovariectomized (Ovx) rats at 60 days. A - Maximum and minimum dF/dt in increasing extracellular calcium concentrations from 0.62 to 3.75 mM. B- Effect of the β-adrenergic agonist isoproterenol in increasing concentrations from 10^-8^ to 10^-2^ M in isolated left ventricle papillary muscles from sham operation (Sham) and ovariectomized (Ovx) rats. Extracellular Ca^2+^ concentration was 0.62 mM. Force (g/g) values are expressed as mean ± S.E.M. *different from sham (*P* < 0.05) using two-way ANOVA and post hoc Tukey test. Number of animals used is indicated.

To determine whether the contractile deficit found in the inotropic response to calcium and isoproterenol was also observed in the activation or relaxation phase of force development, we measured the positive and negative rate of force development (dF/dt) (Figure 4). Positive dF/dt was reduced at 60 days after Ovx in all inotropic interventions assayed. There were no significant differences in negative dF/dt between Ovx and sham groups.

### Estrogen replacement therapy

**Figure 5:**
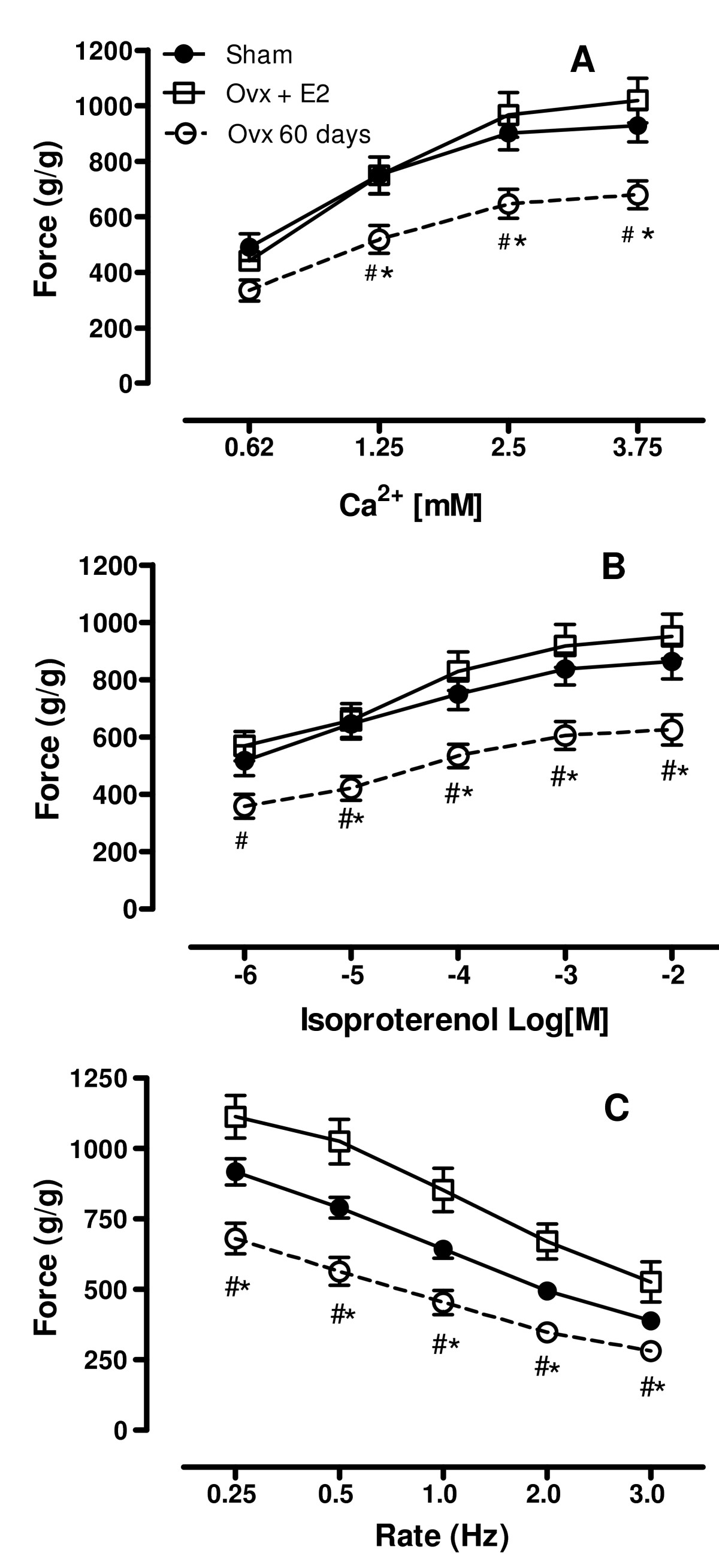
**Estrogen effects on inotropic stimulus**. Effect of estrogen replacement therapy with estradiol valerate on myocardial contractility 60 days after ovariectomy. Estrogen restores inotropic response to extracellular calcium (A) and isoproterenol (B) as well as the rate-isometric force development relationship (C). Force (g/g) values are expressed as mean ± S.E.M. * Ovx n = 8 vs. Sham n = 22, *#* Ovx vs. Ovx + E2 n = 8, different (*P* < 0.05) using two-way ANOVA and post hoc Tukey test.

As previously stated, ovariectomy-induced deficiency in OSH resulted in a significant decrease in inotropic response only after long-term (60 days) hormone deprivation. To determine if estrogen was involved in this effect, ovariectomized rats were treated with estradiol valerate for 8 weeks starting one week after surgery. Estrogen replacement efficiently prevented the decrease in the positive inotropic response to extracellular Ca^2+^ (Figure 5A) and isoproterenol (Figure 5B), and also restored the force-frequency relationship (Figure 5C).

### Western blot analysis of SERCA2a, NCX and PLB

**Figure 6:**
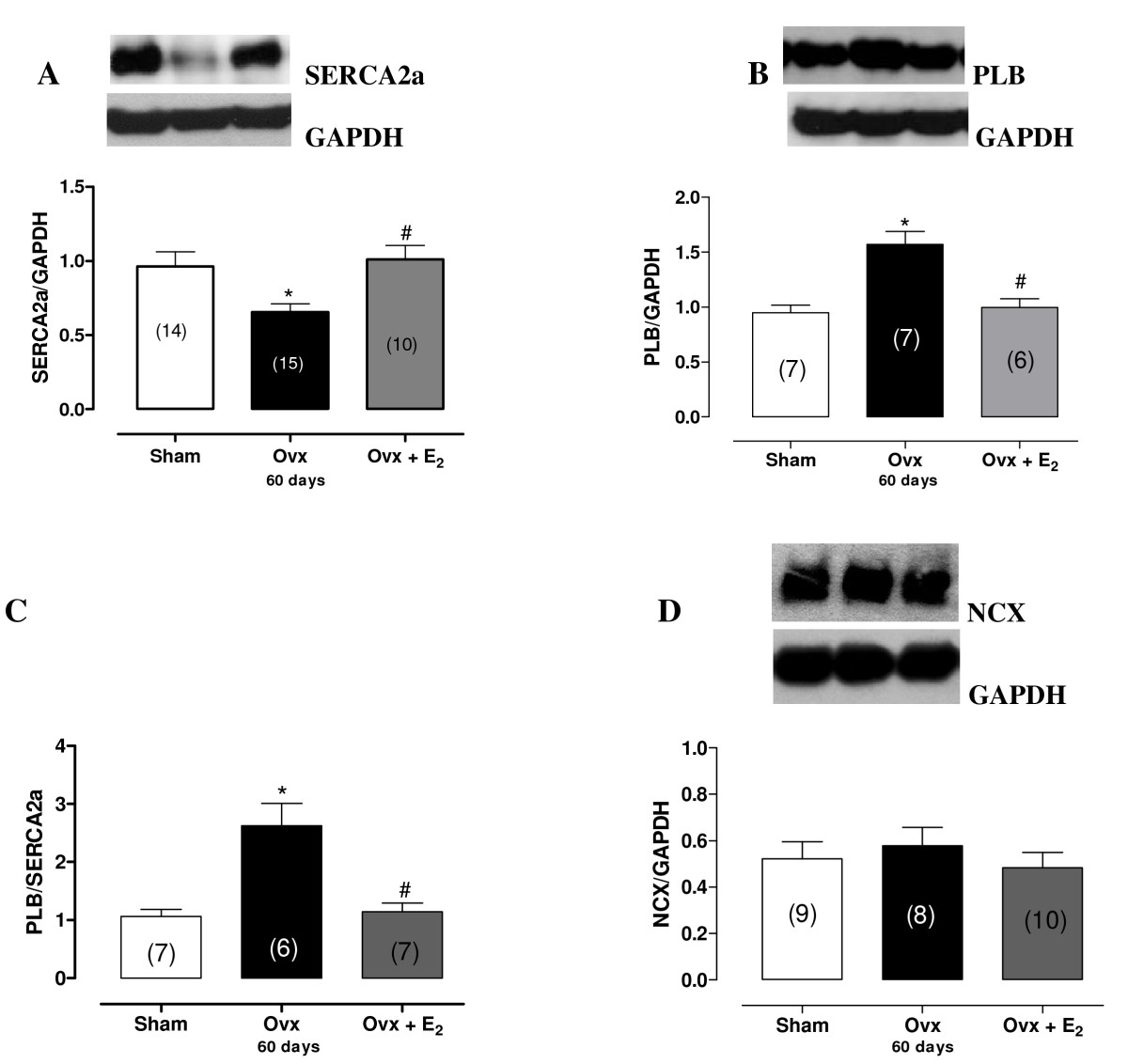
**Calcium handling protein expression**. Densitometric analysis of Western blots for (A) SR Ca^2+^-ATPase (SERCA2a), (B) phospholamban (PLB), (C) SERCA2a/PLB ratio and (D) sodium - calcium exchange (NCX) in hearts from sham, Ovx and Ovx + E_2_ rats. * *p* < 0.05 by ANOVA vs. Sham rats. # *p* < 0.05 by ANOVA vs. Ovx rats. Number of animals used is indicated in parentheses. Representative blots are shown.

**Figure 7:**
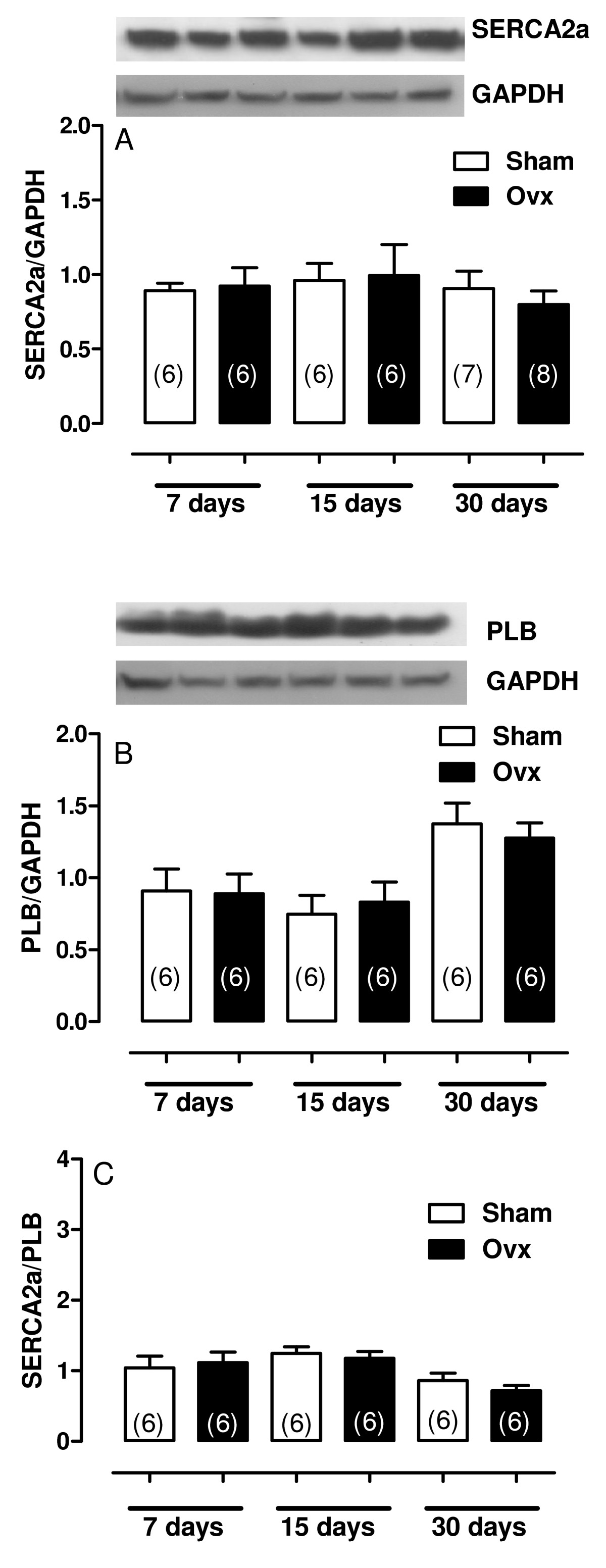
**Calcium handling protein expression**. Densitometric analysis of Western blots for (A) SR Ca^2+^-ATPase (SERCA2a), (B) phospholamban (PLB), (C) SERCA2a/PLB ratio in hearts from Sham and Ovx at 7, 15 and 30 days after surgery. Two way ANOVA. n = 6. Representative blots are shown.

Alterations to cardiac mechanical properties and intracellular calcium homeostasis are dependent on SERCA2a, PLB and NCX. In this study, we examined the role of these proteins in the altered myocardial contractility seen in rats 60 days after ovariectomy. As shown in the Figure 6A and 6B, Ovx altered SERCA2a. Ovx reduced SERCA2a protein expression and increased PLB protein expression in the heart. E2 administration was effective in preventing those changes. Figure 6C demonstrates that PLB was overexpressed by 1.6 fold compared to the sham group. Meanwhile, NCX protein expression remained unchanged (Figure 6D). We also evaluated SERCA2a and PLB in rats at 7, 15 and 30. SERCA2a and PLB did not differ between Sham and Ovx at 7, 15 and 30 days after ovariectomy (Figure 7)

## Discussion

This study examines the effects of OSHs deprivation on cardiac contractility in rats at both short and late-term periods after ovariectomy. Our results demonstrated myocardial contractility was preserved short-term. However, at long-term, deficiency in OSHs reduced cardiac contractility which was associated with changes in expression of key contractile proteins, SERCA2a and PLB. The long-term ovariectomy-induced reduction in myocardial contractility corroborates data previously described [15–18]. The novel finding in this study is that changes in contractility due to ovariectomy were not evident until 60 days after hormone loss. Interestingly, myocardial contractility, analyzed at 7, 15 and 30 days, was preserved during basal conditions and even under positive inotropic stimuli. Besides, at 60 days the Sham group showed an increasing in myocardial contractility response to calcium and Isoproterenol corroborating data previously described [26].

Our results demonstrated that the PLB to SERCA2a ratio was increased at 60 days in the Ovx group by approximately 1.6-fold and was normalized after E2 treatment. The protein expression of NCX remained unchanged. Based on transgenic and gene-targeted mouse model studies, alterations to the PLB to SERCA2a ratio has been suggested to be a major regulator of cardiac contractility [25, 27]. In vivo studies using transgenic mice which overexpress cardiac specific PLB suggested that the "functional stoichiometry" of PLB/SERCA2 is less than 1:1 in native cardiac sarcoplasmic reticulum membranes [27]. Two-fold higher PLB protein levels in these transgenic mice as compared to WT, resulted in greater inhibition of Ca^2+^-ATPase affinity for Ca^2+^, which was associated with decreases in contractility and Ca^2+^ transport in cardiomyocytes.

This suggests that changes observed in SR protein expression at 60 days in Ovx rats may be responsible for the reduced contractility observed. Further, because E2 replacement restored SR protein expression and myocardial contractility, it is plausible that OSHs participate in the long-term regulation of cardiac contractility. In fact, a previous study observed thyroid hormone regulation of SR protein expression and cardiac contractility [28]. These authors also observed enhanced cardiac PLB expression which was associated with decreased rates of cardiac SR Ca^2+^ uptake which is consistent with increased inhibition of the cardiac SR Ca^2+^ pump and decreased contractility in hypothyroid rats.

Long-term studies demonstrated the influence of OSHs on rat cardiac contractility [13] through impaired left ventricular function. Impaired function was characterized by decreases in cardiac output, peak systolic pressure and ejection fraction at all preloads in the hearts of rat ovariectomized before puberty. These contractile changes were further associated with a decrease in myosin ATPase activity. Another study [14] demonstrated the same changes in cardiac function which were reversed by E2 replacement. Further studies reporting changes in intracellular Ca^2+^ homeostasis in cardiomyocytes suggest a possible modulating effect of OSH deficiency on the Ca^2+^ responsiveness of cardiac myofilament activation by induction of myofilament Ca^2+^ hypersensitivity but suppression of maximum myofibrillar ATPase activity [28, 16], which may underlie the cardiac dysfunction observed in OSHs deficiency. These findings support our results. The regulatory role of OSHs in the calcium uptake activity of cardiac SR was also demonstrated in 10 week ovariectomized rat hearts [16]. These authors demonstrated that estrogen and progesterone supplementation were equally effective in preventing changes in ovariectomized hearts.

In our study, 30 days after Ovx, myocardial isometric contractility in response to extracellular Ca^2+^ and β-adrenergic agonist as well as the rate-force development relationship were preserved. In another study, hormone status did not affect levels of SERCA2a, PLB, ryanodine receptor and the mRNA encoding for β_1_-adrenoreceptor at 4 weeks post ovariectomy in rat [17]. Chu et al. further demonstrated that there was no difference in the isoproterenol-elicited increase in developed force though the abundance of β_1_-adrenoreceptor was over 2-fold higher in the Ovx group compared with Sham.

We also conducted our study at an early stage after ovariectomy. Rat hearts were studied at 7 and 15 days post ovariectomy. Clear evidence of OSH absence was observed as early as 7 days after ovariectomy with a close to 3-fold reduction in uterus weight compared to sham animals. In addition, there was an increase in body weight in the Ovx rats at 15 days. However, myocardial contractility was preserved in these animals.

E2 receptor signaling in the cardiovascular system is a complex process that is not completely understood. Its effects on the vascular system have been demonstrated in normal rats [30] and after myocardial infarction [31]. It is well recognized that E2 has genomic and non-genomic actions. The long-term effects of estrogen are mediated by different nuclear hormone receptors, ER α and ER β, which are encoded by different genes and act as ligand-dependent transcription factors [32]. To date, a number of mechanisms have been proposed to decipher the cardio protective effects of E2. E2 alters the expression of ventricular ß_1_-adrenoreceptor [17, 33], intracellular calcium homeostasis related to the L-type Ca^2+^ channel [35], cardiac SR Ca^2+^ uptake [16] and Ca^2+^ sensitivity of cardiac myofilaments in ovariectomized rats [29], which are important mediators of cardiac contractility. Although a cardiac transcriptional regulation of estrogen is well described, there is little direct evidence of membrane cardiac estrogen receptors. Data reporting on the non-genomic signal transduction in cardiomyocytes have demonstrated that 17β estradiol has a negative inotropic effect on guinea-pig single ventricular myocytes by inhibiting ICa and so reducing systolic Ca^2+^ [35].

Furthermore, in isolated rat ventricular cardiomyocytes, it was demonstrated that E2 exerts opposite effects on intracellular myocyte pH, which is associated with pro-and anti-hypertrophic effects [36].

A question raised by the results presented here is whether OSHs affects the myocardium directly or by secondary mechanisms involving other hormones modulated by E2. Also, as contractility was altered so late after loss of OSHs, does E2 act only by genomic mechanisms, or are compensatory mechanisms involved in the short term? Further studies are necessary to clarify these questions. Taken together, our results suggest that changes in Ca^2+^ handling due to abnormal SR function may contribute to reduced myocardial contractility in chronically OSH-deficient rats. Further studies would be beneficial to determine the importance of these findings in humans, as there are significant species-dependent differences in PLB regulation of SERCA2a [37]. Further, long-term rather than short-term estrogen suppression lead to contractile dysfunction which was subsequently reversed with estrogen replacement.

## Conclusions

In conclusion, the most novel aspect demonstrated in this study is the long delay in development of cardiac dysfunction after ovariectomy in young female rats.

## Authors’ original submitted files for images

Below are the links to the authors’ original submitted files for images. Authors’ original file for figure 1 Authors’ original file for figure 2 Authors’ original file for figure 3 Authors’ original file for figure 4 Authors’ original file for figure 5 Authors’ original file for figure 6 Authors’ original file for figure 7
