# Supplementary figures and images for: Myocardial contractility is preserved early but reduced late after ovariectomy in young female rats
Source: Reprod Biol Endocrinol. 2011 Apr 23;9:54. doi: 10.1186/1477-7827-9-54 (PMC3107166; doi:10.1186/1477-7827-9-54)

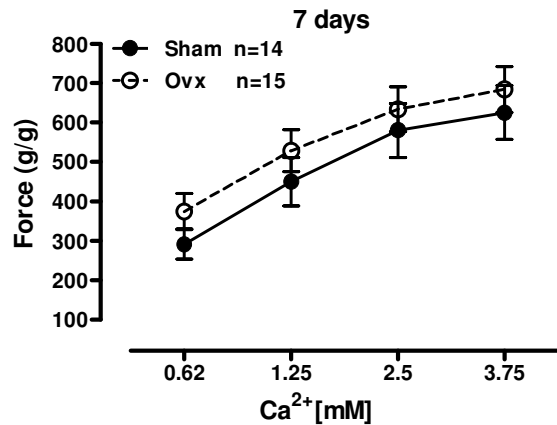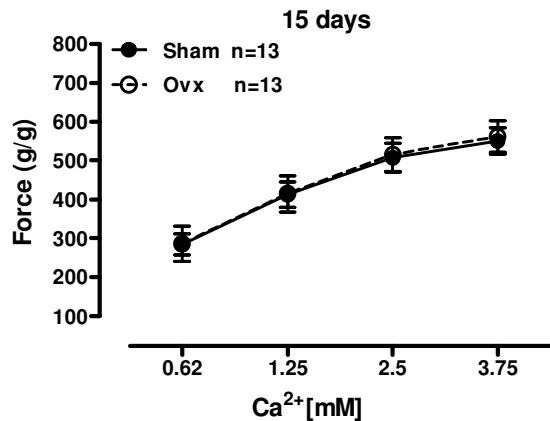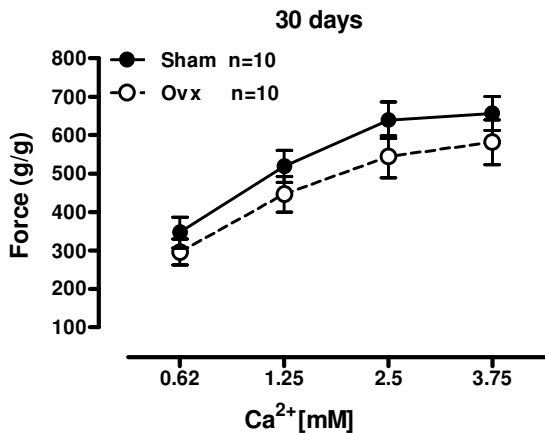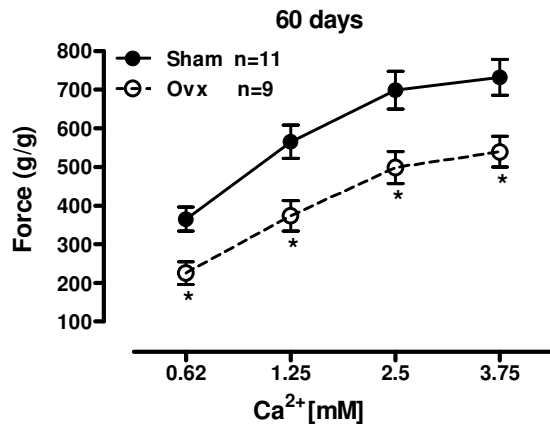

Supplement: Supplementary file 1 — Authors’ original file for figure 1 [file 12958_2011_836_MOESM1_ESM.pdf]

7 days

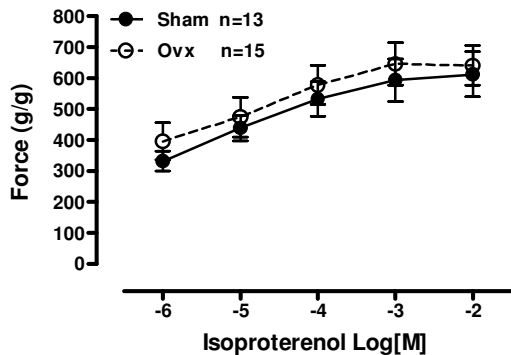

15 days

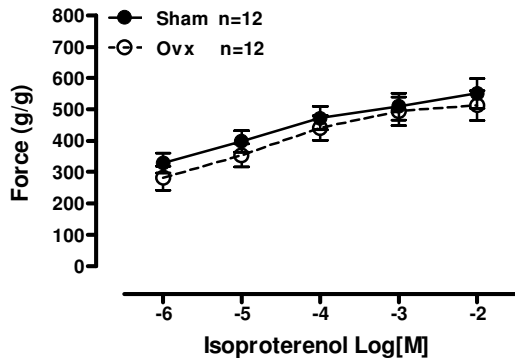

30 days

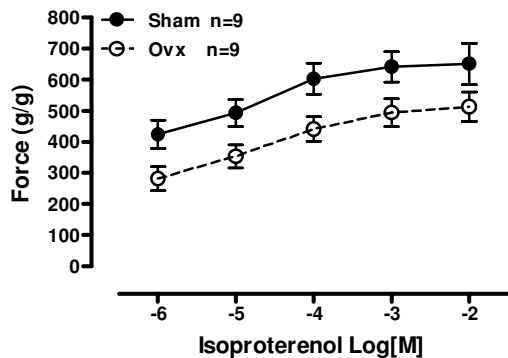

60 days

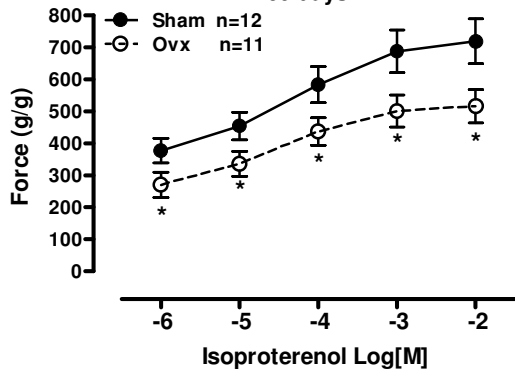

Supplement: Supplementary file 2 — Authors’ original file for figure 2 [file 12958_2011_836_MOESM2_ESM.pdf]

7 days

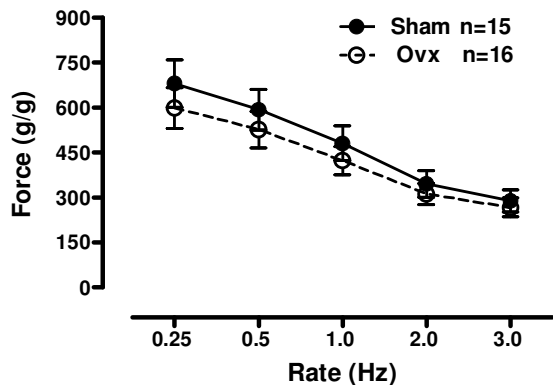

15 days

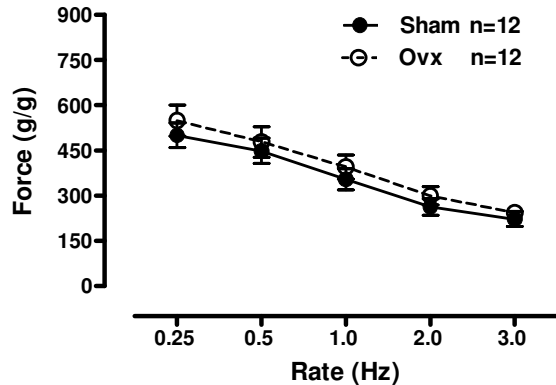

30 days

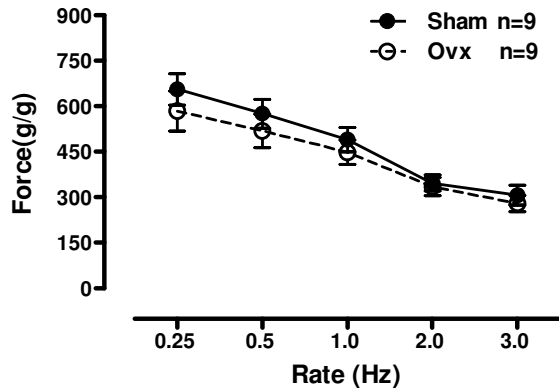

60 days

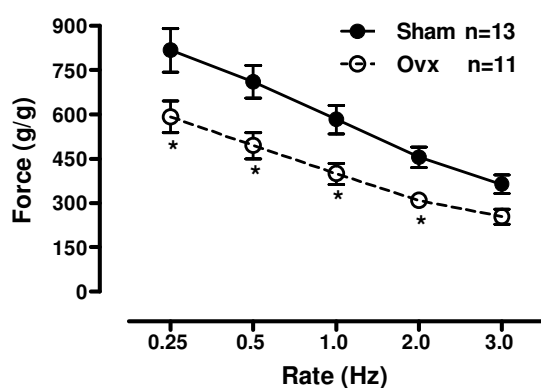

Supplement: Supplementary file 3 — Authors’ original file for figure 3 [file 12958_2011_836_MOESM3_ESM.pdf]

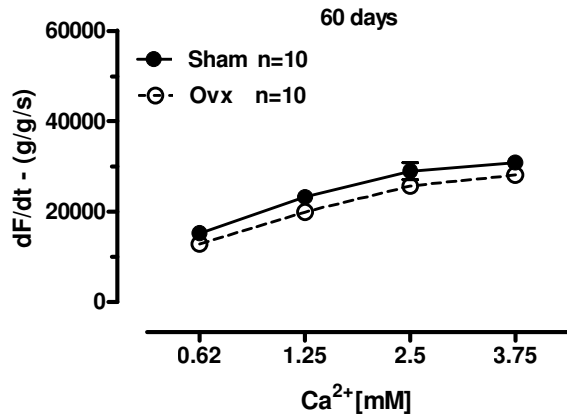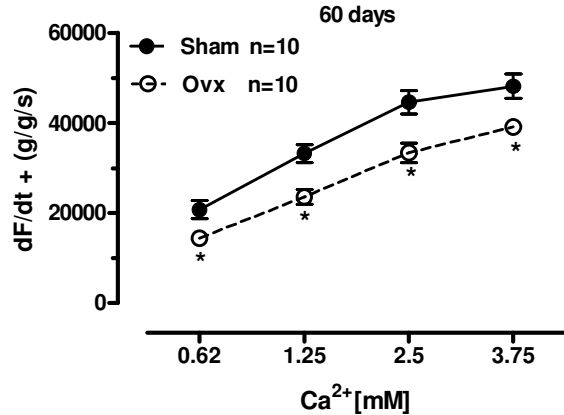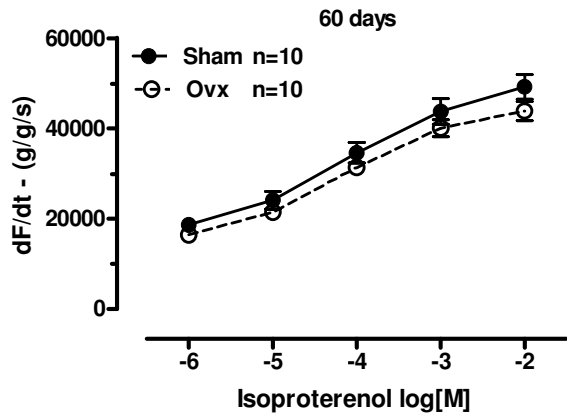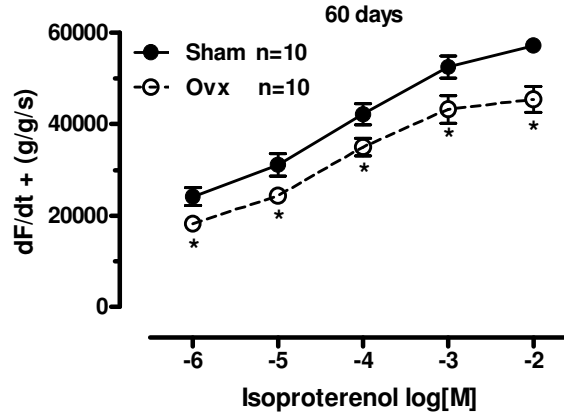

Supplement: Supplementary file 4 — Authors’ original file for figure 4 [file 12958_2011_836_MOESM4_ESM.pdf]

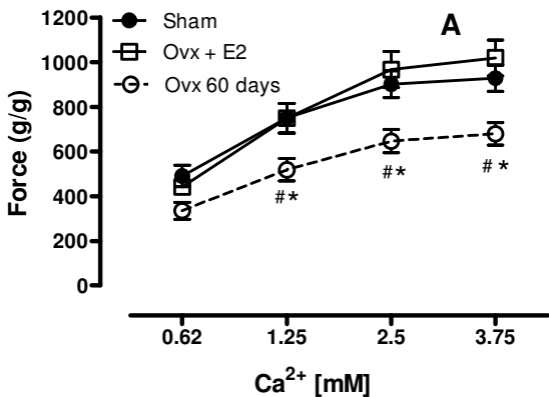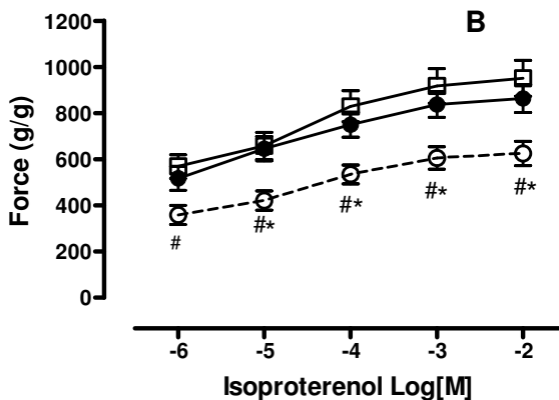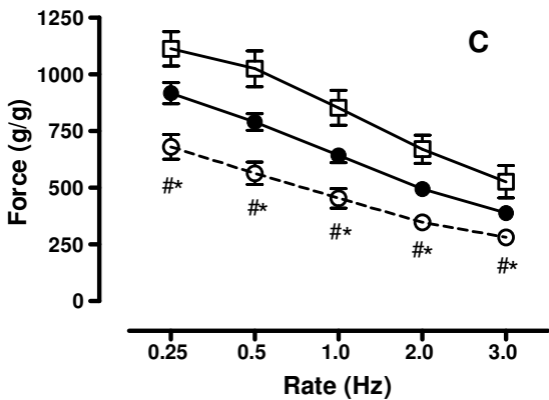

Supplement: Supplementary file 5 — Authors’ original file for figure 5 [file 12958_2011_836_MOESM5_ESM.pdf]

**A**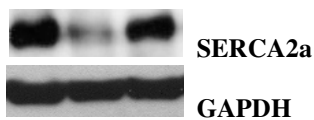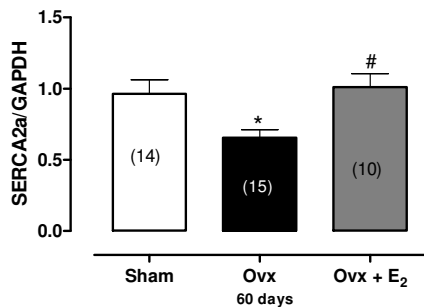**B**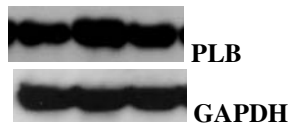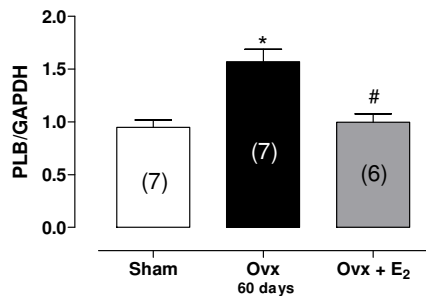**C**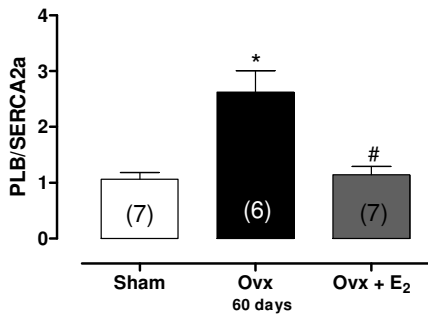**D**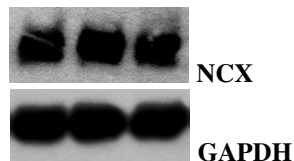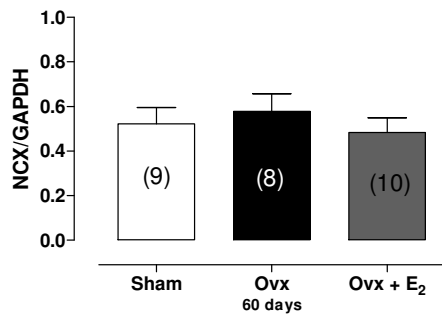

Supplement: Supplementary file 6 — Authors’ original file for figure 6 [file 12958_2011_836_MOESM6_ESM.pdf]

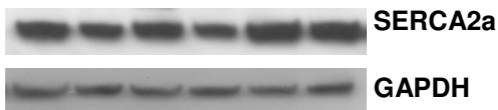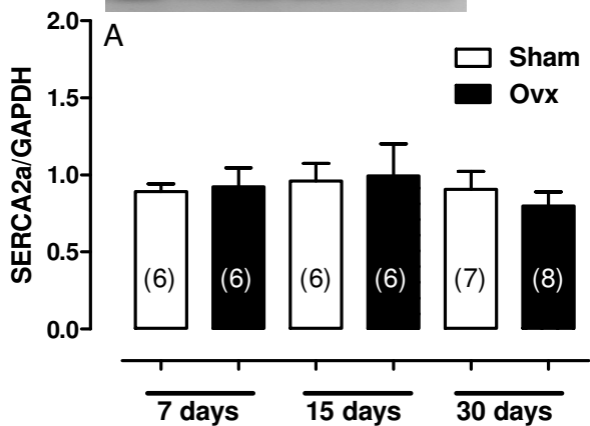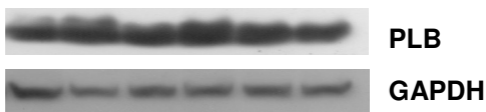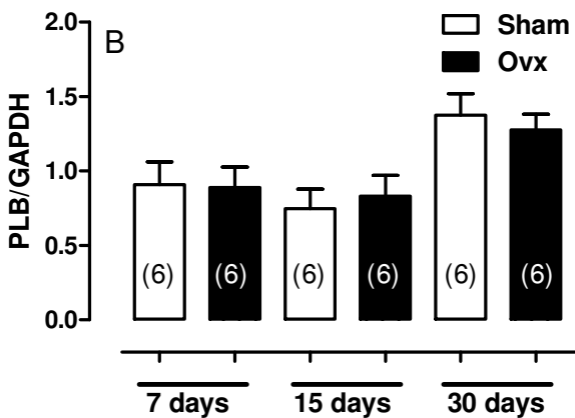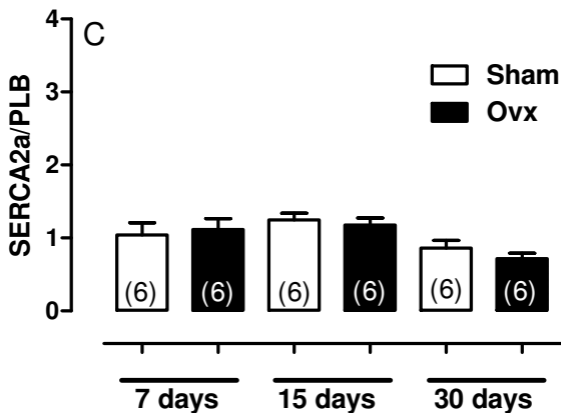

Supplement: Supplementary file 7 — Authors’ original file for figure 7 [file 12958_2011_836_MOESM7_ESM.pdf]
